# P01-023 – Leukocytoclastic vasculitis in a patient with FMF

**DOI:** 10.1186/1546-0096-11-S1-A27

**Published:** 2013-11-08

**Authors:** A Taylan

**Affiliations:** 1 Romatoloji, TEPECIK EĞİTİM VE ARAŞTIRMA HASTANESİ, Izmir, Turkey

## Introduction

A patient with Familial Mediterranean Fever (FMF) in addition to undiagnosed Anklosing Spondylitis (AS) and also having cutaneous leukocytoclastic vasculitis (LV) is presented. Coexistence of FMF with inflammatory disorders including spondyloarthritis and various systemic vasculitides has been reported before. Meanwhile this is the first reported case of FMF, AS, and LV present together.

## Case report

A 35 year old Caucasian man with known diagnosis of FMF was admitted for his cutaneous eruption of both lower limbs. He also had swelling and pain in his right wrist and left knee. Apart from colchicine pills, the patient had no history of recent drug exposure and also infectious symptoms like fever, abdominal pain, and diarrhea,but complaining of inflammatory back pain without diagnosis for 10 years. On examination, there were macular, cutaneous eruptions on both lower legs. In addition to arthritis, the patient also features of AS, such as painful restriction of spinal movements (modified schober 3cm).The remaining of the physical examination was normal.

Laboratory tests revealed hemoglobin of 9.3 g/dL.ESR and CRP were 59 mm/h, 7.27 mg/dL respectively. Radiographs of sacroiliac joint were compatible with bilateral grade IV sacroiliitis. A skin biopsy from tibia showed fibrin deposits, nuclear debris, endothelial swelling and neutrophils disrupting a capillary wall. Immune fluorescence staining was clear for immune deposit. Serum IgA level was 617mg/dl (82-453) and HLA-B27 antigen was positive. ANA, ANCA, and cryoglobulins were undetectable; complement levels and urine examination were in normal limit. Genetic analysis was consistent with compound heterozygote mutation (R202Q/ R726A) at MEFV gene.

Treatment with corticosteroid, sulfosalazine, asemetazin, colchicin was commenced. Almost 10 days after treatment the skin eruptions was fade; arthritis and back pain regressed.

## Discussion

Besides arthritis, several spondyloarthropathic features like sacroiliitis and enthesopathy can be seen in the course of FMF. Sacroiliitis with apophyseal joint involvement without vertebral squaring and bamboo spine characterize FMF-related sera negative spondyloarthropathy, while anterior radiologic involvement of the spine tent to show concomitant presence of AS with FMF. Only apophyseal joints involvement of this case without anterior vertebral column involvement suggests that the lesion of this case was FMF-related.A skin biopsy showing no immune deposit, several urine examination without microscopic hematuria or proteinuria together with lack of GI symptoms and preceding infection all rules out IgA nephropathy and Henoch Schönlein Purpura .Some patients who have taken drugs may develop drug related LV. In this case, absences of history of recent exposure to drugs also discard this possibility Several vasculitic disorders including HSP, PAN, and Protracted febrile myalgia was reported to be associated with FMF . Among the MEFV gene, M694V mutations through high IL-1*β* activity cause a severe form of FMF. To our knowledge, cutaneous vasculitis with immune complex nephritis in a FMF patient was reported by Schlesinger et al [1]. In our case, MEFV mutation and HLA-B27 might have acted as a genetic susceptibility factor for LV development.

## Disclosure of interest

None declared.
